# Time- and Dose-Related Effects of Di-(2-ethylhexyl) Phthalate and Its Main Metabolites on the Function of the Rat Fetal Testis *in Vitro*

**DOI:** 10.1289/ehp.11870

**Published:** 2008-12-01

**Authors:** François Chauvigné, Arnaud Menuet, Laurianne Lesné, Marie-Christine Chagnon, Cécile Chevrier, Jean-François Regnier, Jürgen Angerer, Bernard Jégou

**Affiliations:** 1 INSERM (Institut National de la Santé et de la Recherche Médicale), U625, GERHM, Université Rennes I, Campus de Beaulieu, Rennes, France;; 2 UMR FLAVIC, ENSBANA (Ecole Nationale Supérieure de Biologie Appliquée à la Nutrition et à l’Alimentation), Dijon, France;; 3 ARKEMA France, Département Toxicologie et Environnement, Colombes, France;; 4 Institut und Poliklinik für Arbeits-, Sozial- und Umweltmedizin, Erlangen, Germany

**Keywords:** androgens, anti-Müllerian hormone, endocrine disruption, explant culture, fetal testis, gonocytes, phthalates

## Abstract

**Background:**

Endocrine-disrupting effects of phthalates are understood primarily from *in utero* exposures within the fetal rat testis. Nevertheless, their path of action, dose–response character, and cellular target(s) within the fetal testis are not known.

**Objectives:**

In this study we investigated the effects of di-(2-ethylhexyl) phthalate (DEHP), mono-(2-ethylhexyl) phthalate (MEHP), and several of their metabolites on the development of organo-cultured testes from rat fetus.

**Methods:**

We removed testes from 14.5-day-old rat fetuses and cultured them for 1–3 days with or without DEHP, MEHP, and the metabolites.

**Results:**

DEHP (10^−5^ M) produced a proandrogenic effect after 3 days of culture, whereas MEHP disrupted testis morphology and function. Leydig cells were the first affected by MEHP, with a number of them being inappropriately located within some seminiferous tubules. Additionally, we found a time- and dose-dependent reduction of testosterone. By 48 hr, gonocyte proliferation had decreased, whereas apoptosis increased. Sertoli cell number was unaffected, although some cells appeared vacuolated, and production of anti-Müllerian hormone decreased in a time- and dose-dependent manner. The derived metabolite mono-(2-ethyl-5-hydroxyhexyl) phthalate was the only one to cause deleterious effects to the rat fetal testis *in vitro*.

**Conclusion:**

We hope that this *in vitro* method will facilitate the study of different phthalate esters and other endocrine disruptors for direct testicular effects.

The development of the fetal testis requires complex hormonal regulation and thus forms a highly sensitive detector for hormonal disruption (Sharpe 2006). Phthalate esters are known to be endocrine disruptors, chemicals that alter normal endocrine function and regulation, in particular, during male sex determination and differentiation (Foster 2005; Henley and Korach 2006; Waring and Harris 2005). Di-(2-ethylhexyl) phthalate (DEHP) is the most common chemical additive used in the manufacture of plastics and is consequently distributed widely, for instance, in infant toys, cosmetics, food packaging, and medical devices [Agency for Toxic Substances and Disease Registry (ATSDR) 2002]. Because DEHP is not covalently bound to the polymers, it leaches from the matrix and is endemic to the human environment (Hashizume et al. 2002; Rudel et al. 2003), so humans are constantly exposed to phthalates through oral, dermal, and inhalation routes (Koch et al. 2006; Wormuth et al. 2006). After exposure, DEHP is rapidly hydrolyzed by esterases in the gut, liver, and blood into mono-(2-ethylhexyl) phthalate (MEHP), which is believed to be the active molecule (Gray 1986). MEHP is then hydrolyzed to mono-(2-ethyl-5-hydroxyhexyl) phthalate (5OH-MEHP) and metabolized into mono-(2-ethyl-5-oxohexyl) phthalate (5OXO-MEHP) (Koch et al. 2003). MEHP is also ω-oxidized into mono-(2-ethyl-5-carboxy-pentyl) phthalate (5CX-MEPP) (Koch et al. 2005). The degree of *in utero* exposure has been demonstrated in humans when DEHP and MEHP were found in amniotic fluid, cord blood, and embryonic tissues (Latini et al. 2003; Silva et al. 2004). Moreover, DEHP has been proposed to exert disrupting effects on male reproductive development in a prospective mother–son cohort investigation (Swan et al. 2005) and to alter a number of endocrine parameters in infants through breast-feeding (Main et al. 2006).

In animal models, experimental data have established phthalate toxicity on testicular function in prenatal, neonatal, and post natal rats. Thus, pregnant rats exposed to DEHP or di-*n*-butyl phthalate gave birth to fetuses with reproductive tract defects such as hypospadias, decreased anogenital distance, retained nipples, epididymal agenesis, malformed seminal vesicles, and cryptorchidism (Imajima et al. 1997; Jiang et al. 2007; Rider et al. 2008; Shono et al. 2005). The great majority of these *in vivo* studies were conducted *in utero* and cannot distinguish between direct versus indirect effects via metabolites. In the present study, we assessed the direct effects of DEHP and its metabolites MEHP, 5OH-MEHP, 5OXO-MEHP, and 5CX-MEPP on testis development using an organotypic culture system previously established for the rat (Habert et al. 1991; Lassurguere et al. 2003). This is *in vitro* system displays many developmental events observed *in vivo* (Livera et al. 2006) and supports the independent study of the dose- and time-related effects on somatic and germ cells of fetal testes.

## Materials and Methods

### Animals and sample collection

Pregnant female Sprague-Dawley rats from the indoor breeding colony of GERHM (Research Group on Human and Mammalian Reproduction)–INSERM, U625, and from Elevage Janvier (Le Genest Saint-Isle, Laval, France) were anesthetized by intraperitoneal injection of 40 mg/kg sodium pentobarbital (Sanofi-Synthélabo, Libourne, France) on gestation day 14.5 (GD14.5; day of mating was GD0) according to the French ethics committee of the University of Rennes I. The testes were aseptically removed from the fetuses under a binocular microscope and then immediately explanted *in vitro*. All animals were kept under standard housing conditions and were treated humanely and with regard for alleviation of suffering.

### Culture procedure

Testes were cultured on 0.45 μm Millipore filters (Millipore Corp., Bedford, MA, USA) as previously described (Habert et al. 1991; Lassurguere et al. 2003). Briefly, each GD14.5 fetal testis was removed with the adjacent mesonephros, placed on a filter floating on 0.5mL Gibco M199 medium (Invitrogen, Eragny sur Oise, France) supplemented with 50 μg/mL gentamicin (Life Technologies, Cergy-Pontoise, France) and 2.5 μg/mL Fungizone (Life Technologies) in culture dishes, and incubated in a humidified atmosphere (5% CO_2_ at 37°C) for 24, 48, or 72 hr. One testis from each embryo was cultured with medium containing 0.1% DMSO (Prolabo, Fontenay-sous-Bois, France) as the control and the other containing 10^−4^, 10^−5^, or 10^−6^ M DEHP (purity > 99%; Interchim, Montluçon, France) or MEHP (purity > 99%; Interchim) or 10^−5^ M 5OH-MEHP, 5OXO-MEHP, or 5CX-MEPP (purity > 97%; the MEHP metabolites were prepared at the Institut und Poliklinik für Arbeits). All phthalates were diluted in DMSO to a final concentration of 0.1%. One-half of the volume of the culture medium was refreshed every 24 hr.

In a number of experiments, at the end of the culture period, we added 100 ng/mL ovine leutinizing hormone (LH; NIH. LH S19; 1.01 NIH.LH.S1 U/mg; gift from A.F. Parlow, National Institute of Diabetes and Digestive and Kidney Diseases, Bethesda, MD, USA) or 1 mM 5-bromo-2-deoxyuridine (BrdU; Amersham, Buckinghamshire, UK) to the dishes in 500 μL of fresh medium for 3 hr. Then, the whole explant was fixed for 2 hr in 4% paraformaldehyde in phosphate-buffered saline (PBS) at 4°C, washed in PBS, dehydrated, and embedded in paraffin. To estimate the concentration of the phthalate present within the fetal gonad, testes were incubated with 10^−5^ M radiolabeled phthalates following the same culture procedure described above. We used DEHP-ring-UL-^14^C (^14^C-DEHP; 6.6 mCi/mmol; purity > 98%; Sigma-Aldrich, Saint-Quentin-Fallavier, France) and ^14^C-MEHP (2.2 mCi/mmol; provided by J.P. Cravedi, Institut National de la Recherche Agronomique, UMR 1089, Toulouse, France).

At the end of the culture, testes were separated from filters and both were digested in 1 mL Soluene-350 (PerkinElmer, Courtaboeuf, France) and then incubated at 55°C (2 hr for the testes, overnight for the filters). The digested testes were dissolved in 10 mL Ultima Gold scintillating liquid (PerkinElmer), and radioactivity was counted using a Tricarb 2100 TR scintillation counter (PerkinElmer). These experiments allowed us to calculate that 0.3% of added DEHP and 0.4% of added MEHP, both at 10^−5^ M, were present within the testes after 72 hr of culture. To assess the possible nonspecific effects of the phthalates on the different cellular compartments, we performed a thorough morphologic assessment of the gonads at the different time points of the culture and we directly counted the different cell types. Furthermore, to investigate the specificity of the effects of phthalates on testosterone production, we also tested the possible effects of 10^−5^ M and 10^−4^ M methyl palmitate (Sigma-Aldrich), which possesses the same lipophilic properties as DEHP (Krop et al. 1997; Staples et al. 1997).

### Identification of different cell types

To determine whether DEHP and MEHP caused histopathologic defects in rat fetuses, we immunostained the different testicular cell types using primary rabbit antibodies directed against DDX4/VASA (DEAD-box protein 4) for gonocytes (1:200; Abcam, Paris, France), 3β-hydroxysteroid dehydrogenase (3β-HSD) for Leydig cells (1:500, provided by J.J. Feige, INSERM U878, Grenoble, France), and Wilms tumor gene protein 1 (WT-1; 1:500; Santa Cruz Biotechnology Inc., Santa Cruz, CA, USA) or anti-Müllerian hormone (AMH; 1:1,000; provided by J.Y. Picard, INSERM U782, Clamart, France) for Sertoli cells. The embedded testes were serial sectioned transversally at 5 μm. Deparaffinized sections were rehydrated, washed in PBS, heated for 5 min in 0.01 M citrate buffer (pH 6.0), and incubated for 30 min in PBS with 1% bovine serum albumin as a blocking agent. The slides were incubated overnight at 4°C with the primary antibody, rinsed several times in PBS before 1 hr of treatment with a biotinylated goat anti-rabbit secondary antibody (DAKO, Trappes, France) followed by incubation for 30 min with an avidin-biotin-peroxidase complex [streptavidin-horseradish peroxidase (HRP), 1:500; DAKO]. Staining was visualized with 3,3′-diaminobenzidine (DAB; DAKO) or a 3-amino-9-ethyl carba-zole (AEC) chromogen kit (Beckman Coulter, Villepinte, France). The reaction was stopped by immersion in water; sections were then counterstained with hemalin and mounted.

### Counting of cell types

We counted the immunopositive cells using the Computer-Assisted Stereology Toolbox (CAST) Grid System (Olympus, Copenhagen, Denmark) on a light microscope (Olympus BX S1). At first, we delineated the testis borders at low magnification. Then, we counted the immunopositive cells at 100× using a high-numerical-aperture objective lens on a random fraction comprising 20% of every third section. Counted cells were then multiplied by 3 to obtain the crude cell count (CC). To correct for double counting of a single nucleus found in two sections, we used the Abercrombie formula (Abercrombie 1946):


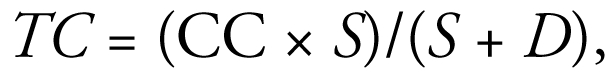


where *TC* is the corrected number of labeled cells per testis, *S* is the thickness of the sections (5 μm), and *D* is the average diameter of the nuclei of labeled cells, obtained by division of the mean nuclear diameter defined on sections by π/4 (Lassurguere et al. 2003). All counts and measurements were performed blind to treatment conditions.

### Gonocyte apoptotic and mitotic indexes

For apoptotic cell staining, we performed terminal deoxynucleotidyl transferase-mediated 2′-deoxyuridine 5′-triphosphate nick end labeling (TUNEL) assays (In Situ Cell Death Detection Kit, POD; Roche Diagnostics, Meylan, France) following a method previously described by Lassurguere et al. (2003) and Livera et al. (2000). Briefly, sections were deparaffinized, rehydrated, and pretreated by microwave boiling in 0.01 M citrate buffer. After washing in PBS, endogenous peroxidase was quenched in 3% hydrogen peroxide/PBS buffer. DNA fragmented in apoptotic cells was labeled by enzymatic incorporation of fluorescein-12-dUTP (Boehringer Ingelheim, Paris, France) at the free 3′ OH-termini of the DNA fragments and exposed to anti-fluorescein antibody conjugated to peroxidase (Boehringer Ingelheim). The apoptotic cells were then revealed by DAB. To restrict the counting of the TUNEL-positive gonocytes, the sections were secondarily incubated with a DDX4/VASA antibody (Abcam), visualized by AEC (Beckman Coulter), and counter-stained and mounted.

We defined a mitotic index by adding 50 μM BrdU (Abcam) to media during the 3 hr before fixation (Lassurguere et al. 2003). BrdU-positive cells were detected by immunohistochemistry. Sections were deparaffinized, rehydrated, and microwave-cooked for 5 min in 0.01 M citrate buffer (pH 6.0). After washing in PBS, slides were incubated successively in 3% H_2_O_2_/PBS for 15 min, in 2N HCl at 37°C for 20 min, and in 0.07 M NaOH for 10 min. Sections were then rinsed in PBS and incubated overnight with anti-BrdU monoclonal antibody (DAKO). We detected the antibody using a peroxidase-linked anti-mouse IgG cell proliferation kit (Amersham, Les Ulis, France) followed by incubation with streptavidin-HRP and finally visualization with DAB. The BrdU incorporation index was defined by counting at least 500 gonocytes specifically recognized by immunostaining with the DDX4/VASA antibody visualized with AEC, as described above.

### Testosterone assay

The medium of each culture was recovered every 24 hr and stored at −80°C until a testosterone radioimmunoassay could be performed (Testosterone Direct RIA kit; Beckman Coulter, Villepinte, France). Each sample was assayed in duplicate, without prior extraction.

### AMH assay

We used an AMH enzyme-linked immunosorbent assay (ELISA) kit (DSL-10-14400; Diagnostic Systems Laboratories Inc., Cergy Pontoise, France) according to the manufacturer’s instructions to determine the AMH level secreted by testes in the media. Each sample (20 μL of daily half-medium recovered) was assayed in duplicate, without prior extraction; the limit of detection was 0.006 ng/mL.

### Statistical analysis

Wilcoxon signed rank and Wilcoxon Mann-Whitney tests were performed on paired and unpaired data, respectively, for the two-sample tests. We used non parametric analysis of variance (ANOVA) for the dose–response experiments. We used SAS/STAT software (version 9.1; SAS Institute Inc., Cary, NC, USA) to perform all statistical analyses.

## Results

### MEHP decreases gonocyte number

**Figure 1:**
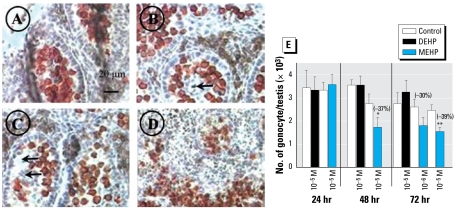
Effect of DEHP and MEHP on testicular histology (*A*–*D*) and the total number of gonocytes (*E*) after 72 hr of culture beginning on GD14.5 (see “Materials and Methods” for experimental details). (*A*) Histology of nonexposed (control) fetal testis. (*B, C, D*) Effect of 10^−6^ M (*B*), 10^−5^ M (*C*), or 10^−4^ M (*D*) MEHP on the histology of seminiferous tubules in fetal rat testes after 72 hr in culture. Gonocytes were immunostained with DDX4/VASA antibody, revealed by AEC (red), and Leydig cells were immunostained with 3β-HSD antibody, revealed by DAB (brown). Scale in (*A*) also applies to (*B–D*). Arrows in (*B*) and (*C*) indicate holes and degenerative gonocytes within seminiferous tubules. In (*D*), seminiferous tubules were not recognizable. (*E*) Effect of DEHP and MEHP on the total number of gonocytes. Responses to DEHP and MEHP were measured by comparing one control testis (DMSO-treated) with the contra lateral testis cultured in medium containing the tested factor. Values are mean ± SE of 3–7 fetuses in at least two independent experiments. The numbers in parentheses indicate the percent decrease relative to the corresponding control. *p < 0.05, and **p < 0.01 by Wilcoxon signed rank tests performed on paired data.

The gross morphology of seminiferous tubules was disturbed after treatment with 10^−6^ M, 10^−5^ M, and 10^−4^ M MEHP (Figure 1A–D) but not with DEHP (data not shown). At 10^−4^ M MEHP, we saw a global disorganization of the testis morphology (Figure 1D). The staining intensity of DDX4/VASA immunolabeling of gonocytes in itself did not appear to be affected by MEHP. However, total numbers of gonocytes in MEHP-exposed testes were significantly reduced in a time- and dose-dependent manner (Figure 1E).

### MEHP induces a decrease of gonocyte mitotic index and an increase of gonocyte apoptosis

**Figure 2:**
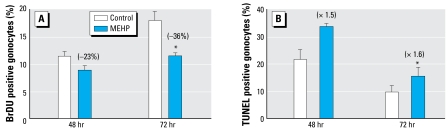
Effect of 10^−5^ M MEHP on the mitotic index and apoptosis of gonocytes. (*A*) Quantitative analysis of BrdU incorporation into gonocytes at 48 and 72 hr of culture measured as the percentage of BrdU-positive gonocytes in at least 500 cells (*n* = 4 fetuses at each time point). (*B*) Quantitative analysis of apoptotic gonocytes measured as the percentage of TUNEL-positive gonocytes in at least 500 cells (*n* = 4 fetuses at 48 hr and *n* = 6 fetuses at 72 hr). The numbers in parentheses indicate the percent decrease (*A* ) or the factor of increase (*B*) relative to the corresponding control. *p < 0.05 by Wilcoxon signed rank tests on paired data.

When cultured in the presence of 10^−5^ M MEHP, the percentage of BrdU-labeled gonocytes started to decline (−23%) at 48 hr and had significantly decreased (−36%) at 72 hr (Figure 2A). Furthermore, the number of apoptotic gonocytes in cultures treated with 10^−5^ M MEHP, as revealed by counting TUNEL-positive cells, was increased by factors of 1.5 and 1.6 at 48 hr and 72 hr, respectively (Figure 2B).

### MEHP disrupts Sertoli cell function but has no effect on Sertoli cell number

**Figure 3:**
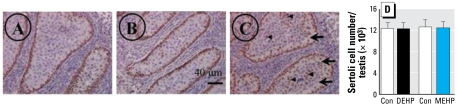
Effect of DEHP and MEHP on Sertoli cell morphology (*A–C*) and the total number of Sertoli cells (*D*) in cultured testes. Con, control. (*A–C*) One testis from each fetus was cultured in control medium (*A*) and the other in medium supplemented with DEHP (*B*) or MEHP (*C*) at 10^−5^ M. Sertoli cells were immunostained with WT-1 antibody, revealed by DAB (brown). In the MEHP-treated testis (*C*), some Sertoli cells appeared vacuolated (arrows) and aggregated (arrowheads). (*D* ) Sertoli cells were counted after 3 days of culture using systematic random sampling accomplished by the CAST-grid stereotaxic system. Values are mean ± SE of 7–8 fetuses from at least two independent experiments analogous to A–C. No significant difference was found in the Sertoli cell number by Wilcoxon signed rank tests of paired data.

**Figure 4:**
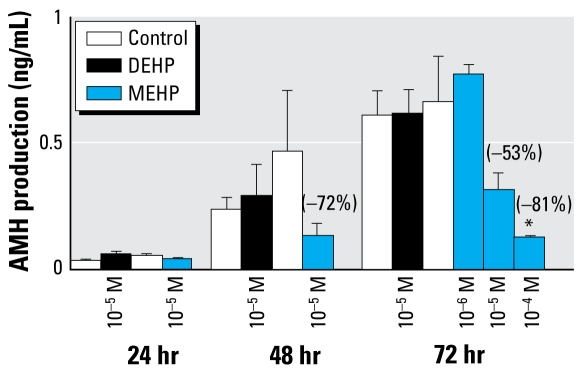
Effects of DEHP (10^−5^ M) and MEHP (10^−6^ M, 10^−5^ M, or 10^−4^ M) on Sertoli cell function revealed by *in vitro* AMH secretion by GD14.5 fetal rat testes cultured for 24, 48, or 72 hr and measured by ELISA (see “Materials and Methods” for experimental details). Values are mean ± SE of 3–4 fetuses; values in parentheses indicate the decrease relative to control. In the dose–response study at 72 hr, four groups of testes (from different dams and formed randomly) were exposed to 10^−5^ M DEHP or 10^−6^ to 10^−4^ M MEHP, and compared with corresponding control values. A non-parametric ANOVA on paired data with repeated measures indicated that in controls, a statistically significant increase of AMH production occurred over time, and a statistically significant decrease of AMH production occurred with 10^−5^ M MEHP exposure (*p*< 0.05). *p < 0.05 by Wilcoxon signed rank tests of paired data.

Neither DEHP nor MEHP altered the total number of Sertoli cells (Figure 3D), and nuclei diameter was unchanged (mean ± SE diameter: control, 6.41 ± 0.22 mu;m; DEHP, 6.45 ± 0.20 μm; MEHP = 6.49 ± 0.18 μm; *n* = 30). However, 10^−5^ M MEHP exposure produced Sertoli cell foci within the seminiferous cords (Figure 3C, arrowheads), and some appeared vacuolated (Figure 3C, arrows); this was not observed after DEHP exposure (Figure 3B). When we used immunohistochemistry to assess AMH expression *in situ*, we observed no differences in the labeling of the Sertoli cells (data not shown). Despite this, at 10^−5^ M, MEHP did inhibit AMH production at 48 hr (−72%; Figure 4). We observed a dose-dependent inhibitory effect of MEHP at 72 hr, with no effect at 10^−6^ M, but inhibition of 53% and 81% at 10^−5^ M and 10^−4^ M, respectively. We observed a time-dependent increase of AMH production between 24 and 72 hr of culture (Figure 4). These pheno types were all MEHP specific, with no effects occurring with DEHP treatment.

### DEHP and MEHP have no effect on Leydig cell number

**Figure 5:**
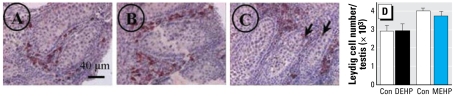
Effect of DEHP and MEHP on the location (*A–C*) and number (*D*) of Leydig cells after 72 hr of culture beginning on GD14.5 (see “Materials and Methods” for experimental details). Con, Control. (*A–C*) Leydig cells were immuno stained with 3β-HSD antibody and revealed by AEC (red) after treatment with control (*A*), 10^−5^ M DEHP (*B*) or 10^−5^ M MEHP (*C*). After MEHP exposure, we observed a low but consistent number of Leydig cells abnormally located within the seminiferous tubules (*C*, arrows). The scale in (*A*) also applies to (*B–C*). (*D* ) Leydig cells were counted using systematic random sampling accomplished by the CAST-grid stereotaxic system. Values are mean ± SE of 4 fetuses from at least two independent experiments. No significant difference in number was found using Wilcoxon signed rank tests.

Neither DEHP nor MEHP had any effect on 3β-HSD staining intensity (Figure 5A–C), Leydig cell number (Figure 5D), or the Leydig cell nuclear diameter (control, 5.51 ± 0.16 μm; DEHP, 5.54 ± 0.17 μm; MEHP, 5.28 ± 0.11 μm; *n* = 30). However, we consistently observed a number of cells marked by 3β-HSD–positive staining as Leydig cells within seminiferous tubules in MEHP-exposed gonads (Figure 5C, arrows).

### DEHP stimulates testosterone production, whereas MEHP inhibits it

**Figure 6:**
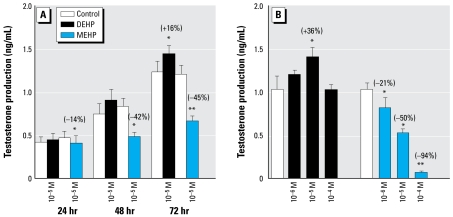
Time course (*A*) and dose response (*B*) showing effects of DEHP and MEHP on basal testosterone secretion by fetal rat testes cultured beginning on GD14.5. Testosterone was measured by radioimmuno assay. (*A*) For the time course, one testis from each fetus was cultured in the control medium, and the contralateral testis was cultured in medium containing DEHP or MEHP. One-half of medium was changed every 24 hr. (*B*) Effects of increasing concentrations of DEHP and MEHP on basal testosterone production after 72 hr of culture. In this case, the control and phthalate-exposed testes did not originate from the same fetuses; the testes of different fetuses and from different dams were assigned at random. In (*A*), values are mean ± SE of 18–34 fetuses from at least three independent experiments; in (*B*), values are mean ± SE of 7–14 testes from at least two independent experiments. The values in parentheses indicate the percentage of decrease or increase relative to the corresponding control. *p < 0.05; and **p < 0.01 by Wilcoxon signed rank tests on paired data in (*A*) and nonparametric ANOVA on unpaired data in ( *B*).

**Figure 7:**
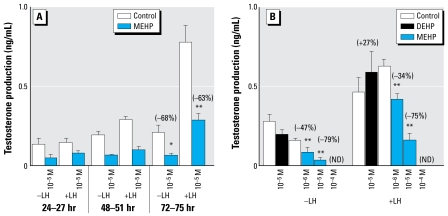
Time course (*A*) and dose response (*B*) showing effects of MEHP and DEHP on basal and LH-stimulated testosterone secretion by fetal rat testes cultured beginning on GD14.5. (*A*) For the time course, one testis from each fetus was cultured for 27, 51, or 75 hr in control medium or in medium containing MEHP, with media changed every 24 hr. For the last 3 hr of the culture period, medium was supplemented with (+LH) or without (−LH) 100 ng/mL LH (24–27 hr, 48–51 hr, and 72–75 hr). (*B*) Effects of 10^−5^ M DEHP and increasing concentrations of MEHP on basal and LH-stimulated testosterone production after 72 hr of culture. In this case, the control and phthalate-exposed testes did not originate from the same fetuses; the testes of different fetuses and from different mothers were assigned at random. In (*A*), values are mean ± SE of 6–18 fetuses from at least two independent experiments; in (*B*), values are mean ± SE of 3–10 testes from at least two independent experiments. The values in parentheses indicate the percentage of decrease (−) or increase (+) relative to control. *p < 0.05; and **p < 0.01 by Wilcoxon signed rank tests on paired data in (*A*) and nonparametric ANOVA on unpaired data in (*B*). ND, not detectable.

A progressive increase in testosterone production was typical of the testis organoculture (Figures 6A, 7A). In the presence of 10^−5^ M MEHP, testosterone production was slightly but significantly decreased (−14%) at 24 hr (Figure 6A). From 48 hr of culture onward, 10^−5^ M MEHP prevented the age-related increase in testosterone production (Figure 6A). The MEHP-decreased testosterone production corresponded to 42% at 48 hr (Figure 6A). By 72 hr, although 10^−5^ M DEHP slightly increased testosterone levels (*p* < 0.05), exposure to 10^−5^ M MEHP significantly decreased these levels by 45% (Figure 6A). In another series of experiments, we confirmed the stimulation of testosterone by DEHP at 72 hr (Figure 6B). Furthermore, we observed a dose-dependent decrease in testosterone production using MEHP, with the lower concentration (10^−6^ M) reducing the hormone levels by 21% (Figure 6B). When basal testosterone production was assessed in successive 3-hr time periods (24–27 hr, 48–51 hr, 72–75 hr) instead of the 24-hr time periods, as presented in Figure 6A and B, we always observed an inhibitory effect of 10^−5^ M MEHP on basal testosterone levels, although this inhibition was significant only for the 72–75 hr time period (*p* < 0.02 at 72–75 hr vs. *p* < 0.06 at earlier times; Figure 7A). The presence of LH in the culture media revealed an increased ability of the fetal gonad to secrete testosterone along the culture period. Interestingly, the fetal gonad also appeared to respond to LH in terms of testosterone production as early as 48 hr (*p* < 0.05; Figure 7A). In the presence of 10^−5^ M MEHP, the LH-induced testosterone levels were always lower (*p* < 0.06 at 27 and 51 hr; *p* < 0.001 at 75 hr; Figure 7A). Although data in Figure 7B confirm the inhibitory effects of MEHP on basal and LH-induced testosterone production described above (Figure 6B), we also found a dose–response relationship in the MEHP inhibition of testosterone. This MEHP-induced decrease in testosterone production was partially reversible, as shown by passage to phthalate-free medium (data not shown). Also, methyl palmitate at concentrations up to 10^−4^ M had no effect on testosterone levels (data not shown), which indicates that the phthalate-induced effects of MEHP most likely did not result from the intrinsic lipophilic nature of these compounds.

### Effects of MEHP and its oxidized metabolites

**Figure 8:**
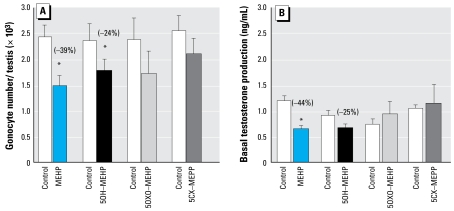
Effects of DEHP metabolites MEHP, 5OH-MEHP, 5OXO-MEHP, or 5CX-MEPP (10^−5^ M) on the number of gonocytes (*A*) and on testosterone production (*B*) in fetal rat testes cultured for 72 hr beginning on GD14.5. One-half of medium was changed every 24 hr for both (*A*) and (*B*). (*B*) Testosterone was measured by radioimmuno assay. In (*A*), values are mean ± SE of 5–7 fetuses from at least two independent experiments; in (*B*), values are mean ± SE of 6–20 animals from at least two independent experiments. Values in parentheses indicate the percent decrease relative to corresponding control. *p < 0.05 by Wilcoxon signed rank tests on paired data.

At 10^−5^ M, MEHP and 5OH-MEHP significantly decreased gonocyte number by 39% and 24%, respectively. Furthermore, basal testosterone production was decreased by 44% (*p* < 0.001) and 25%, respectively, after treatment with MEHP or 5OH-MEHP (Figure 8A,B). In contrast, neither 5OXO-MEHP nor 5CX-MEHP produced such deleterious effects (Figure 8A).

## Discussion

Based on a search of PubMed (U.S. National Library of Medicine, Bethesda, MD, USA) using the terms “phthalate” and “testis,” we found that < 11% of the approximately 300 references used *in vitro* experimental approaches. However, using an *in vitro* system to study the effects of phthalates on the fetal testis has a number of important advantages. In particular, it is possible to investigate direct effects of these compounds using controlled concentrations. The kinetics of action and primary cellular target(s) within the gonads are more easily determined using an *in vitro* approach, and they validate its use in addition to, or in place of, the classical *in utero* approach.

In the present study we used a rat organotypic culture system that supports normal differentiation of the fetal gonad during at least 3 days (Livera et al. 2006). Although this system has previously been validated for toxicology purposes using estradiol and diethylstil bestrol (Lassurguere et al. 2003), at first we were unable to see any effect of DEHP or MEHP (data not shown). These results were in agreement with similar attempts to use this system (Hallmark et al. 2007; Li and Kim 2003; Stroheker et al. 2005). However, when we modified the culture conditions by changing half of the culture media daily instead of a complete daily change/replacement (Lassurguere et al. 2003), we then observed a number of direct phthalate-induced effects on the cultured fetal gonad.

In another series of experiments, we determined that DEHP is not metabolized under our culture conditions (data not shown). This indicates that, between GD14.5 and GD17.5, the rat fetal gonad does not express the esterase and cytochrome P450 enzyme systems that are involved in the metabolism of phthalate esters. Furthermore, this also indicates that the effects of these compounds reported in the present study were most likely due to the compound tested rather than metabolites. In the *in utero* experiments reported by Stroheker et al. (2006), most of the DEHP was metabolized, and only a very small proportion of it (2–5%) was able to cross the placental barrier and be detected in the GD18 fetal testis. A central observation of our *in vitro* study is that DEHP caused no deleterious effects. This finding is compatible with previous experiments establishing that MEHP is the active metabolite of DEHP and is involved in the testicular disruption observed *in utero* (Foster et al. 2001).

We found that 10^−4^ M MEHP reduced testosterone levels to the threshold of detection and disorganized the testis to such a degree that cell counting was impossible. This is why we chose to perform more experiments using the 10^−5^ M concentration. This level allowed us to explore the sequence of action of MEHP on different cell types. By tracking ^14^C-DEHP and ^14^C-MEHP in different compartments of our culture systems, we determined that at 10^−5^ M, only 0.3–0.4% of these two phthalates were present in the testicular fetal explants under our culture conditions, which corresponded to a concentration of about 2,500 μg/L. Because of this and because we consistently saw antiandrogenic effects with the lower dose of MEHP (10^−6^ M), we consider the active concentrations of MEHP found in our study to be within an order of magnitude of the concentrations found in recent studies in the plasma from pregnant women [1,150 μg DEHP/L and 680 μg MEHP/L (Latini et al. 2003)] or in neonate cord blood [2,050 μg DEHP/L and 520 μg/L MEHP (Latini et al. 2003)].

At 10^−5^ M MEHP, we observed mislocalization of Leydig cells within the seminiferous tubules, between the Sertoli cells. This finding matches *in utero* studies in the rat (Fisher et al. 2003; Mahood et al. 2006). Although the total number of Leydig cells did not vary, basal Leydig cell testosterone production appeared markedly reduced in the MEHP-exposed cultures in a time- and dose-dependent manner. These findings suggest that the antiandrogenic properties displayed by certain phthalates administered *in utero* actually result from direct action on fetal Leydig cell biology. We also found LH-stimulated testosterone production to decrease in a time- and dose-dependent manner. However, the intrinsic relative amplitude of the response of Leydig cells to LH was unchanged by MEHP. This indicates that the antiandrogenic activity of MEHP occurs separately from Leydig cell receptivity to LH. Interestingly, the cultured fetal gonads were responsive to LH from GD15.5 to GD16.5, which is compatible with earlier studies establishing that both the transcript for LH receptor and LH receptor itself were first detected by GD16.5 *in situ* (Majdic et al. 1998; Zhang et al. 1994). LH is normally expressed by the fetal pituitary only from GD16.5 and is not detected in serum before GD18 (El-Gehani et al. 1998; Migrenne et al. 2001). Ultimately, this finding establishes the competence of the organoculture system insofar as it supports responsiveness to LH and marks the beginning of LH responsiveness in rat testes. Interestingly, that the inhibition of testosterone levels induced by MEHP was not due to a nonspecific cytotoxic effect was established not only by the careful morphologic assessment of our study (histologic examinations, Leydig cell counts), but also by our results showing that methyl palmitate, a compound known to have the same lipophilic properties as phthalates (Krop et al. 1997; Staples et al. 1997), had no effect on testosterone production in the fetal gonad assay.

Unexpectedly, DEHP increased testosterone production at a concentration of 10^−5^ M. This proandrogenic ability of DEHP has rarely been reported. Inhalation of DEHP has been reported to stimulate testosterone production in prepubertal rats in a time- and dose-dependent manner (Kurahashi et al. 2005). Likewise, DEHP seems to exert a positive effect on hepatic steroidogenesis (Fan et al. 2003; Wong and Gill 2002). According to a number of studies in rats (Barlow et al. 2003; Lehmann et al. 2004; Plummer et al. 2007), some genes involved in cholesterol uptake, transport to mitochondrion, and steroidogenesis are down-regulated after *in utero* administration of di-*n*-butyl phthalate, another phthalate known to exert *in utero* effects similar to those of DEHP (Howdeshell et al. 2007). This indicates that the antiandrogenic effects of phthalates most probably result from their action at different transcriptional levels downstream of the LH receptor. In humans, 75% of the DEHP ingested is metabolized and excreted in urine within 2 days (Koch et al. 2005), although the resulting metabolites clear at different rates: 5OH-MEHP and 5OXO-MEHP clear quickly, whereas 5CX-MEPP takes longer (Koch et al. 2005). In urine from children, Becker et al. (2004) found concentrations of 5OH-MEHP and 5OXO-MEHP to be 8.0-fold and 6.2-fold higher than those of MEHP. This suggests that fetuses may experience greater exposure to these metabolites than to MEHP itself. Of the five phthalate esters tested here, DEHP appeared pro androgenic, whereas MEHP and, to a lesser extent, its first metabolite, 5OH-MEHP, were antiandrogenic.

We found that MEHP reduced the total number of gonocytes as early as 48 hr in culture, but phthalate-induced gonocyte apoptosis has also been described in the rat *in utero* (Ferrara et al. 2006) and in humans *in vitro* (Lambrot et al. 2009). Our finding stands in contrast to those of Li and Kim (2003), as these authors saw no effect of 100 μM MEHP on the number of gonocytes in testes after 3 days of culture of GD13 and GD18 rat testes. This could be due to differences in the age at which the gonads were collected and cultured [GD13–GD16 by Li and Kim (2003) vs. GD14.5–GD17.5 here], because the critical windows of sensitivity of the rat fetal testis to phthalate and to the classical antiandrogen flutamide have been determined to be GD15–GD18 (Carruthers and Foster 2005) and GD15.5–GD17.5 (Welsh et al. 2008), respectively. We determined that the decrease in gonocyte number in MEHP-exposed testes resulted from a combination of decreased proliferation and increased apoptosis. The mechanism behind this decrease remains unknown. Other toxicants that decrease gonocyte number, such as flutamide (Mylchreest et al. 1999), which antagonizes androgen signaling, produce distinctly different phenotypes. *In vitro* analyses of human fetal testes (obtained between 7 and 12 weeks of gestation) have revealed no effect of MEHP on basal or LH-stimulated testosterone production (Lambrot et al. 2009). Similarly, Hallmark et al (2007) did not observe any effect of 10^−3^ M monobutyl phthalate on human fetal testes obtained between 15 and 20 weeks of gestation Whether this absence of MEHP effect on human testes reflects a difference between rat and human biology or simply that the 7- to 12-week or 15- to 20-week windows do not correspond to the phthalates sensitive stage in humans *in vitro* remains to be elucidated.

Much remains to be elucidated to understand the mechanism(s) by which MEHP induces both an increase in apoptosis and a decrease in proliferation rate for gonocytes. Multiple but complementary pathways could be involved. For instance, recent *in vitro* studies using cultured Sertoli cells showed that MEHP induces the expression by Sertoli cells of solubilized Fas ligand, which binds to Fas, a germ-cell surface receptor able to trigger germ cell apoptosis (Boekelheide et al. 1998; Giammona et al. 2002). Another report proposes that gonocyte apoptosis may be directly induced by MEHP via the transcription of Fas ligand (Yao et al. 2007). Other pathways, such as the tumor necrosis factor-related apoptosis-inducing ligand-related pathway (McKee et al. 2006) and the nuclear factor κ-B pathway (Rasoulpour and Boekelheide 2005) could also be involved in MEHP-induced germ cell apoptosis. Further investigation will be required to determine which elements mediate the MEHP effect on the gonocytes and more generally on the testis.

In the present study, neither DEHP nor MEHP had any effect on Sertoli cell number. This result corroborates *in utero* studies showing that Sertoli cell number is not affected in phthalate-exposed GD17 testes (Scott et al. 2008). However, vacuolization of these cells after exposure to 10^−5^ M MEHP for 3 days is similar to the pheno type previously reported by Borch et al. (2006) for GD20–GD21 rat testis exposed *in utero* to DEHP and other phthalates. Certainly Sertoli cell function was affected because MEHP inhibited AMH production in a time- and dose-dependent manner. AMH immunolabeling in a similar study (Li and Kim 2003) was not affected by MEHP in fetal gonads exposed to the same phthalate concentration although, as noted above, those fetal gonads were explanted at a younger age than in the present study. Furthermore, when we undertook immuno histochemical labeling of Sertoli cells with AMH antibody, we also were unable to observe quantitative variation of AMH levels owing to the poor sensitivity of that assay. Ultimately, with higher levels of MEHP (10^−4^ M), Li and Kim (2003) observed a decrease in AMH labeling. This is the same concentration at which we observed an 81% decrease in AMH production and dramatically altered morphology of the testis. Lambrot et al. (2009) also recently found an MEHP-induced decrease in the mRNA expression of AMH by human fetal testis *in vitro* at 10^−4^ M. Whether the MEHP-induced alteration of Sertoli cell morphology and function is mediated by effects on testosterone, a reduced gonocyte population, or a combination remains to be established.

In conclusion, our study demonstrates the facility of an *in vitro* system, the fetal gonad assay, as an effective assay for the effects of MEHP and other potential endocrine disruptors. This alternative to *in utero* study elucidates direct dose -and time-dependent effects of phthalates. Using this system, we found that deleterious effects of MEHP first occurred in Leydig cell function, followed by a concomitant decrease in the germ cell pool and in Sertoli cell function. Most important, we demonstrated that the actual active dose of MEHP corresponds to a concentration within an order of magnitude of the phthalate concentrations found in human biological fluids. We are now using the fetal gonad assay to investigate the precise mechanisms by which MEHP disrupts steroidogenesis.
